# Potential Drug-Drug Interactions in Hospitalized Medical Patients: Data From Low Resource Settings

**DOI:** 10.7759/cureus.17336

**Published:** 2021-08-20

**Authors:** Khalid Rashid, Yahya Khan, Farrukh Ansar, Aamir Waheed, Muhammad Aizaz

**Affiliations:** 1 Internal Medicine, James Cook University Hospital, Middlesbrough, GBR; 2 Department of Medical Education, Pak International Medical College, Peshawar, PAK; 3 Medicine, Northwest School of Medicine, Khyber Medical University, Peshawar, PAK; 4 Internal Medicine, Northampton General Hospital, Northampton, GBR; 5 Community Health Sciences, Peshawar Medical College, Peshawar, PAK

**Keywords:** drug-drug interactions, polypharmacy, low resource setting, prescription audit, adverse outcomes

## Abstract

Introduction

Adverse events related to Drug-Drug Interactions (DDIs) are among the few common reasons for hospitalization worldwide; however, they can be prevented with an efficient patient-centered system. Different mechanisms have successfully limited the prevalence of DDIs in developed countries. There are limited data regarding DDIs from limited-resource settings. Furthermore, there is no cost-effective system that has shown promising results in preventing them in this setting. This study aims to assess the frequency of potential DDIs in a low-resource setting and to check its association with different factors such as poly-pharmacy and demographics.

Methods

Through this cross-sectional study, drug charts of patients admitted to a medical unit in November 2019 were analyzed using a structured questionnaire. A list of drugs co-prescribed to each patient was entered into the Medscape Drug Interaction checker to calculate the frequency and severity of potential DDIs.

Results

The mean age of patients was 49 years, and on average, seven drugs were prescribed to each patient. Among 100 analyzed prescriptions, 400 potential DDIs were identified with a mean of 4±5.42 per patient. According to Medscape interaction checker classification, 2 DDIs were contraindicated, 28 were serious, 246 required close monitoring, and 124 were minor. The most frequently encountered drug interaction was "spironolactone with furosemide." There was a significant correlation of the occurrence of potential DDIs with increased numbers of prescribed drugs.

Conclusion

Our patient population was prescribed more drugs per patient than calculated in other settings. Poly-pharmacy is an independent risk factor for DDIs. Lastly, advancing age exposes patients to poly-pharmacy, and therefore, they are at a higher risk of developing DDIs.

## Introduction

Drug safety is an essential part of treatment and patient health, particularly in a hospital setting. A crucial aspect of drug safety is the drug-drug interactions (DDIs) that can lead to severe side effects and affect therapeutic efficacy. Adverse drug events (ADEs) are defined as injuries resulting from medical intervention related to a drug [1-3]. They are unwanted consequences of drug administration and are the leading cause of the increase in hospital stay and mortality worldwide, including in developed countries [2-4]. Most ADEs are due to interactions between different drugs, as most patients admitted to the hospital are on poly-pharmacy [2,3]. These DDIs have been commonly reported in high-risk populations such as the elderly and patients with multiple comorbidities. The main reason for this distribution is that the probability of poly-pharmacy increases with age, and also, most chronic diseases are treated with multidrug regimens [5]. Studies have also shown a deficiency in the prescription process and a need for more sensitivity in healthcare professionals regarding DDIs [5-7].

Developed countries have reported success in curtailing the incidence of DDIs by introducing various measures and initiatives. For instance, Halkin et al. reported a significant reduction in DDIs after introducing digital prescription methods and interaction screening across pharmacies and hospitals [6]. Garg et al. [7] have also reported a significant reduction in ADEs due to computerized clinical decision support systems (CDSSs). Other studies have also supported the role of interventions in preventing ADEs, including organizational modifications and educational measures [7-9].

Mousavi and Ghanbari [10] have reported a high prevalence of potential DDIs in the hospital setting in developing countries. Similar results have also been published by Ismail et al. [11] and others [12-15]. However, data that examine the prevalence of potential DDIs in a low-resource setting is very scarce. Therefore, a thorough study for evaluating potential DDIs is essential to assess the prevalence of potential and preventable drug interactions and risk factors associated with them. This would be helpful for designing and implementing efficient and cost-effective measures to prevent DDIs in these settings. This study aims to calculate the number of potential DDIs in the in-patient setting, and determining their risk factors.

## Materials and methods

Our study was a retrospective cross-sectional study conducted at a 125-bed tertiary teaching hospital with both medical and surgical in-patient facilities. Before the initiation of our study, formal written approval was taken from the Ethical Review Board (number: PRM/21/14376). Our study population included all the patients admitted consecutively to the Internal Medicine department from November 1, 2019 to November 30, 2019.

Prescription charts from medical records of all these patients were consulted after the permission of the hospital administration. A total number of 104 drug charts were analyzed, however, four were not included in the analysis as these patients were only prescribed one drug during their short stay. Therefore, data from 100 drug charts were included in the study population.

Patients demographics, including age and gender, were noted, and a list of drugs prescribed to every patient was generated. Subsequently, potential DDIs between co-prescribed drugs were checked using Medscape Drug Interaction Checker, and all the possible DDIs were identified. The potential DDIs were classified into four categories: contraindicated, avoid or use alternative/serious, monitor closely, and minor. The correlation of different factors with DDIs, including age, gender, and the total number of drugs prescribed, were analyzed using Statistical Package For the Social Sciences (SPSS) version 27.0 software (IBM Corp., Armonk, NY).

The objectives of this study were to calculate the frequency of potential DDIs through prescription analysis among the in-patient population of a teaching hospital and to determine the risk factors associated with these potential DDIs.

## Results

Out of 100 enrolled patients in our study sample, 52 were male, and 48 were female. The mean age of patients was 49.52±17.5, ranging from 13 to 84. Age distribution categories are shown in Table 1. The total number of drugs prescribed was 694, ranging between 2 and 15 per patient, with a mean of 6.93±2.94. Moreover, 20% of the patients were prescribed 10 or more drugs on their prescription. Important baseline characteristics are shown in Table 1. Overall, 400 DDIs were identified in our study sample ranging between 0 and 30 per prescription, and the mean DDIs for each patient was 4±5.42. At least one drug interaction was found in prescriptions of 77% of the patients. We identified one DDI in 23 prescriptions, two DDIs in 13 prescriptions, three and four DDIs in four and eight prescriptions, respectively, while 12% of the patients in our sample were exposed to 10 or more DDIs. As per the criteria used in our study, two DDIs fall in the category of contraindicated, 28 (serious), 246 (monitor closely), and 124 (minor) (Table 2). Our results revealed that the most common DDI was spironolactone with furosemide, whereas other interactions of furosemide, spironolactone, aspirin, esomeprazole with other drugs were frequent. The most common DDIs are reported in Table 3.

**Table 1:** Baseline characteristics of patients

Characteristics	Frequency, N (%)
Gender	
Male	52 (52)
Female	48 (48)
Age (mean ± SD)	49.52±17.5
<20	6 (6)
21–40	26 (26)
41–60	44 (44)
61–80	21 (21)
81–100	3 (3)
Prescribed drugs per patient	
<3	1 (0.2)
3–7	62 (45.3)
>7	37 (54.3)
DDIs found in the number of patients	
1–3	40 (40)
4–7	22 (22)
>7	15 (15)

**Table 2:** Severity of DDIs DDI: drug-drug interactions.

Severity of DDIs	Number of DDIs	DDIs present in the number of prescriptions
Contraindicated DDIs	2	1
Serious DDIs	28	17
Monitor closely DDIs	246	55
Minor DDIs	124	61

**Table 3:** Important and common pairs of drug interactions

Interaction	Frequency	Severity (category)	Potential adverse outcome
Spironolactone with furosemide	20	Monitor closely	Serum potassium imbalance
Aspirin with furosemide	8	Monitor closely	Serum potassium imbalance and pharmacodynamic antagonism
Aspirin with spironolactone	8	Monitor closely	Increase serum potassium
Albuterol with furosemide	6	Monitor closely	Pharmacodynamic synergism and hypokalemia
Carvedilol with spironolactone	6	Monitor closely	Increase serum potassium
Metoclopramide with acetaminophen	6	Minor	Aspirin levels rise in blood
Metronidazole with acetaminophen	4	Minor	Hepatic enzyme CYP2E1 metabolism affected
Esomeprazole with cyanocobalamin	4	Minor	Decreases GI absorption
Rifampin with isoniazid	3	Serious	Risk of isoniazid toxicity
Esomeprazole with clopidogrel	2	Serious	Esomeprazole decreases the effects of clopidogrel by affecting hepatic enzyme CYP2C19 metabolism
Ciprofloxacin with theophylline	2	Serious	Ciprofloxacin increases the effect of theophylline which may result in cardiac arrest or seizure
Linezolid with formoterol	1	Contraindicated	Risk of acute hypertensive episode
Linezolid with albuterol	1	Contraindicated	Risk of acute hypertensive episode

Correlation analysis showed that poly-pharmacy was significantly correlated with increased chances of DDIs with Pearson correlation r = 0.628 and a p-value of <0.001. However, other correlation statistics were not significant (p-value >0.05). Multinomial regression analysis was done, which suggested that the male population was more prone to potential DDIs (OR= 3.92, CI: 1.35-11.41, p-value=0.012). Similarly, patients prescribed more than seven drugs had higher odds of experiencing a DDI (OR= 4.40 CI: 1.26-15.34, p-value=0.020; Table 4).

**Table 4:** Potential DDIs and their associations with the risk factors DDIs: drug-drug interactions.

Variable	Drug interactions		P-value	OR (95% CI)
	Present	Absent		
Gender				
Male	45	7	0.012	3.92 (1.35–11.41)
Female	32	16		Reference
Prescribed drugs per patient				
3–7	44	16		Reference
>7	33	4	0.020	4.40 (1.26–15.34)
Age				
<20	4	2	0.144	0.202 (0.024–1.72)
21–40	20	6	0.420	0.535 (0.117–2.44)
41–60	33	11	0.451	0.595 (0.154–2.29)
61–80	20	4		Reference

## Discussion

ADEs significantly increase patient morbidity and mortality and increases the length of hospital stay and healthcare costs. DDIs are one of the causative factors for ADEs, with Pirmohamed et al. reporting that an estimated16.6% of all adverse drug reactions are caused by DDIs [16]. Drug interactions are preventable, but their adverse effects are among the primary reasons for hospital admission and associated complications.

The present study evaluated the presence of potential DDIs in the Internal Medicine ward of the index hospital. A total of 400 DDIs were identified; the mean DDIs for each patient was four, with at least one potential DDI in 77% of the patients (overall prevalence). This result is similar to a study done in Iran, which reported at least one potential DDI in 86.2% of the patients [10]. Another study from Pakistan reported at least one DDI in 52.8% of the study sample [11], while a study conducted in India found the prevalence of potential DDIs in admitted patients to be 30.67% [17]. Studies from the USA and Europe show a prevalence rate of 25% and 46%, respectively [18,19]. The mean DDIs per patient were found to be four in the current study, which is lower than an Iranian study showing an average of 7.6 DDIs per patient [10]. However, our average number of DDIs was relatively higher than few other studies showing an average of 1.2 and 1.4 DDIs per patient [20,21].

This comparison demonstrates that DDIs are common in various clinical settings and require immediate attention. However, every drug interaction is not fatal particularly in critical patients where a multi-drug regimen may be necessary, and few drugs interactions are unavoidable. However, careful monitoring of these patients is required. Therefore, identifying the severity of each DDI is necessary to evaluate the clinical significance and appropriate management. In the present study, the DDIs were divided into four categories; 0.5% of the DDIs were "Contraindicated," 7% were "Serious," 61.5% were in the "Monitor Closely" category, and 31% of the potential DDIs were "Minor." This shows that DDIs with serious adverse effects were less frequent in our study sample. This has also been shown by other studies, such as reported by Egger et al. [21] and others [10,11].

Our analysis showed that a multidrug regimen was significantly associated with increased chances of DDIs. Different studies conducted worldwide bolster our evidence by reporting the same results [11,22]. Poly-pharmacy is a known factor for increased risk of potential DDIs [23]. Although there is no consensus on the definition of poly-pharmacy, the most commonly used definition is using five or more drugs daily [24]. Various studies have shown that potential DDIs are frequent when patients receive prescriptions with multiple drugs [23]. DDIs cause adverse effects and therapeutic inefficiency in many cases, with subsequent poor control of the ailment under treatment [18]. In our study, the mean number of drugs prescribed was 6.93. A study from Northwest Ethiopia reported a mean of 5.59 drugs per prescription [20], while a Brazilian study reported a mean of 5.6 per prescription [25]. The current study shows that poly-pharmacy was significantly correlated with increased chances of DDIs, as shown by other studies.

As evident from our results, patients with advancing age are at greater risk of being prescribed a higher number of regular medications. Similar results have been shown by a study conducted in an almost identical setting by Ismail et al. [11].

As the population is getting older, more people with complex multisystem illnesses such as diabetes and hypertension are prescribed multiple drugs. Most of these drugs are for long-term use and require regular review by pharmacists or clinicians. This concept is often termed deprescribing [26], which is defined by Reeve et al. as the withdrawal process of an inappropriate medication, supervised by a healthcare professional to manage poly-pharmacy and improve outcomes [26]. This term also encompasses a reduction in the dose of the medication in addition to suspending the inappropriate medication [27].

However, this is a demanding task in settings with no proper follow-up. Moreover, in our settings, there is no concept of ward pharmacists reviewing patient's medication while they are inpatient. As a result, a significant number of patients end up taking unnecessary drugs. This practice puts them at risk of developing side effects of that drug and its potentially harmful interactions with other medications.

Reeve et al. analyzed the feasibility of a patient-centered deprescribing system for patients with unnecessary prescriptions of proton pump inhibitors (PPI). It was proved to be efficient in a small size sample of 57 [28]. Moreover, Moorehouse used a pilot model known as the Palliative and Therapeutic Harmonization (PATH) for making complex medical decisions for the older population based on their frailty assessment and cognition. PATH was shown to be effective in this study population of 200 [29].

There are other similar interventions designed to reduce polypharmacy-related adverse events in the elderly population. However, no standard universal tool can be implemented in all settings and is proven effective in large-scale settings. Therefore, keeping in mind the economic burden of this problem, an international body (International Group for Reducing Inappropriate Medication Use and Polypharmacy (IGRIMUP)) was formulated to slow down the adverse effects of this imminent epidemic. They have published their proposed strategies and recommendations to reduce inappropriate drug use and poly-pharmacy. Lastly, they have urged for an urgent and integrated approach to enlist inappropriate medication prescription as the leading global target of the highest priority [30].

Limitations

Due to a lack of sufficient local data, it was not possible to calculate an accurate sample size, and therefore, it was calculated based on a time period of one month. Second, presenting diagnosis and associated comorbidities were not included while collecting the data as that would have added to the significance of our analysis and results.

## Conclusions

To conclude, poly-pharmacy is associated with a higher frequency of potential DDIs. Patients in developing countries are prescribed a more significant number of regular medications than in developed countries. As a result, they are more exposed to ADEs. Moreover, elderly patients are likely to be prescribed a higher number of regular-use drugs than the younger population.

There is a need for an efficient and cost-effective mechanism for preventing DDIs in low-income countries. This can be achieved by incorporating technology into prescription practice and by involving pharmacists in reviewing the medications for admitted patients. These steps have been shown to effectively reduce DDIs in developed countries.
